# CRISPR/Cas9-mediated ablation of *elovl2* in Atlantic salmon (*Salmo salar* L.) inhibits elongation of polyunsaturated fatty acids and induces Srebp-1 and target genes

**DOI:** 10.1038/s41598-019-43862-8

**Published:** 2019-05-17

**Authors:** Alex K. Datsomor, Nikola Zic, Keshuai Li, Rolf E. Olsen, Yang Jin, Jon Olav Vik, Rolf B. Edvardsen, Fabian Grammes, Anna Wargelius, Per Winge

**Affiliations:** 1 Norwegian University of Science and Technology, Department of Biology, Trondheim, N-7491 Norway; 2 Institute of Marine Research, Bergen, N-5817 Norway; 3 Norwegian University of Life Sciences, Department of Animal and Aquacultural Sciences, N-1432 Ås, Norway; 4 BioMar AS, Trondheim, Norway

**Keywords:** CRISPR-Cas9 genome editing, Gene expression, Metabolic engineering, Fatty acids

## Abstract

Atlantic salmon can synthesize polyunsaturated fatty acids (PUFAs), such as eicosapentaenoic acid (20:5n-3), arachidonic acid (20:4n-6) and docosahexaenoic acid (22:6n-3) via activities of very long chain fatty acyl elongases (Elovls) and fatty acyl desaturases (Fads), albeit to a limited degree. Understanding molecular mechanisms of PUFA biosynthesis and regulation is a pre-requisite for sustainable use of vegetable oils in aquafeeds as current sources of fish oils are unable to meet increasing demands for omega-3 PUFAs. By generating CRISPR-mediated *elovl2* partial knockout (KO), we have shown that *elovl2* is crucial for multi-tissue synthesis of 22:6n-3 *in vivo* and that endogenously synthesized PUFAs are important for transcriptional regulation of lipogenic genes in Atlantic salmon. The *elovl2*-KOs showed reduced levels of 22:6n-3 and accumulation of 20:5n-3 and docosapentaenoic acid (22:5n-3) in the liver, brain and white muscle, suggesting inhibition of elongation. Additionally, *elovl2*-KO salmon showed accumulation of 20:4n-6 in brain and white muscle. The impaired synthesis of 22:6n-3 induced hepatic expression of sterol regulatory element binding protein-1 (*srebp-1*), fatty acid synthase-b, *Δ6fad-a*, *Δ5fad* and *elovl5*. Our study demonstrates key roles of *elovl2* at two penultimate steps of PUFA synthesis *in vivo* and suggests Srebp-1 as a main regulator of endogenous PUFA synthesis in Atlantic salmon.

## Introduction

The health benefits of fish oil particularly eicosapentaenoic acid (20:5n-3) and docosahexaenoic acid (22:6n-3) are well documented in many studies and has been linked to the prevention of inflammatory and cardiovascular diseases in humans^1^. Humans however have limited capacity for endogenous synthesis of 20:5n-3 and 22:6n-3^2^ and therefore dietary supplementation of preformed 20:5n-3 and 22:6n-3 remains the best way to meet the requirements in humans. Fishes are primary sources of omega-3 long chain polyunsaturated fatty acids (n-3 LC-PUFAs) in the human food chain^3^ and with stagnant wild capture fisheries, farmed fish including Atlantic salmon now provide increasing proportion of the required n-3 LC-PUFAs for human consumption^4^. Traditionally, formulation of diet for carnivorous farmed fish like Atlantic salmon, (*Salmo salar* L.), relies on high levels of fish oil (FO) and fish meal (FM)^5^. However, the steady increase in Atlantic salmon aquaculture has led to increased substitution with vegetable oils (VOs)^6–8^ which are often characterized by high levels of the C_18_ PUFAs, linoleic acid, 18:2n-6 or linolenic acid, 18:3n-3. Furthermore, these oils are deficient in 20:5n-3 and 22:6n-3^9^. Although VOs have been shown to be promising alternatives to FO for Atlantic salmon ^10–13^, results clearly show limited capacity to produce n-3 LC-PUFAs from 18:3n-3^8^. Accordingly, fish fed VOs have increased levels of 18:3n-3 and 18:2n-6 and decreased content of 20:5n-3 and 22:6n-3, reducing the health-promoting effects of salmon to the human consumer^14^.

Biosynthesis of LC-PUFAs in vertebrates involves alternating steps of desaturation and elongation of the C_18_ PUFAs, 18:3n-3 and 18:2n-6. Synthesis of 20:5n-3 is achieved by Δ6 desaturation of 18:3n-3 to produce 18:4n-3 which is elongated to 20:4n-3 by very long chain fatty acyl elongase 5 (*elovl5*) followed by Δ5 desaturation. Biosynthesis of 22:6n-3 involves two further elongation steps to 24:5n-3, a second Δ6 desaturation to 24:6n-3 followed by a chain-shortening step via peroxisomal β-oxidation through the so called “Sprecher pathway”^15^. A more direct pathway for 22:6n-3 biosynthesis involves elongation of 20:5n-3 to docosapentaenoic acid (22:5n-3) followed by Δ4 desaturation to 22:6n-3, but this pathway may not exist in Atlantic salmon^16,17^. Synthesis of arachidonic acid, 20:4n-6 requires the same set of enzymes, and involves Δ6 desaturation of 18:2n-6 to 18:3n-6 which is elongated to 20:3n-6 followed by Δ5 desaturation^18^. The capacity for LC-PUFA biosynthesis in a species depends on the complementary roles of fatty acyl desaturases (Fads) and very long chain fatty acyl elongases (Elovls). Genes encoding Atlantic salmon fads (*Δ6fad-a*, *Δ6fad-b*, *Δ6fad-c* and *Δ5fad*) and elovls (*elovl5a*, *elovl5b*, *elovl2* and *elovl4*) have been cloned and functionally characterized through heterologous expression in yeast, *Saccharomyces cerevisiae*^19–22^. Salmon *elovl5a* and *elovl5b* elongate C_18_ and C_20_ PUFAs with low activity towards C_22_ ^20,22^, whereas *elovl2* and *elovl4* efficiently convert C_20_ and C_22_ PUFAs^19,22^. Additionally, heterologous expression in *S. cerevisiae* showed that salmon *Δ6fad-a*, *Δ6fad-b*, and *Δ6fad-c* have Δ6 desaturation activity towards 18:3n-3 and 18:2n-6 ^20,21^, while *Δ5fad* predominantly has Δ5 desaturation activity mainly towards C_20_ PUFAs with low level Δ6 activity towards C_18_ PUFAs^20^.

The expression of Atlantic salmon *fad* and *elovl* genes has been shown to be under nutritional regulation, responding to changes in dietary fatty acid composition^21,22^. Results from feeding trials showed upregulation of *elovl2* and *elovl5b* transcripts in the liver of VO-fed salmon compared to FO-fed fish^22^. Similarly, increased expression of *Δ6fad-a* and *Δ6fad-b* was observed respectively in the liver and intestine (pyloric caeca) of Atlantic salmon fed VO^21^. PUFAs exert their effects on transcriptional regulators, particularly Srebp-1 (Sterol regulatory element-binding protein 1)^23,24^ via transcriptional and post-transcriptional mechanisms^23^. The promoter of a member of Srebp family, Srebp-1c, has been shown to contain liver X receptor-alpha (Lxr-alpha) response element and was identified as the primary site mediating n-3 PUFA-dependent regulation of Srebp-1c^23^. Accordingly, 20:5n-3 and in particular 22:6n-3 has been shown to suppress transcription of Srebp-1c by preventing trans-activation of Lxr-alpha in rat hepatocytes^23^. Additionally, treatment of Atlantic salmon established head kidney cell line, SHK-1 with 20:5n-3 and 22:6n-3 downregulated *srebp-1* and reduced expression of target genes, such as fatty acid synthase (*fasn*), *Δ6fad-a* and *Δ5fad*^25^. A detailed understanding of the molecular mechanisms of endogenous LC-PUFA synthesis as well as nutritional and transcriptional regulation in Atlantic salmon will require *in vivo* functional studies. In this study we have generated CRISPR/Cas9 mediated partial knockouts of *elovl2* and analysed the effects on biosynthesis of LC-PUFAs when the salmon are fed two different feed formulations, including one with reduced content of LC-PUFAs. To further support the findings and detect other changes induced by the partial knockout of *elovl2*, a transcriptional analysis by RNAseq was performed. Our results show that 20:5n-3, 20:4n-6 and 22:5n-3 are the main *in vivo* substrates of Elovl2 and show that it is an important elongase needed for 22:6n-3 synthesis in the liver, white muscle and brain. Furthermore, our data suggest that aside dietary LC-PUFAs, endogenously synthesized LC-PUFAs play key roles in transcriptional regulation of hepatic lipogenic genes in Atlantic salmon, most likely through Srebp-1.

## Results

### Generation of *elovl2* KOs and growth performance

Atlantic salmon with indels in the coding region of *elovl2* alleles, (referred to as *elovl2* KOs in this manuscript), were generated by CRISPR/Cas9 as previously described in Atlantic salmon^26^. Three CRISPR constructs targeting exon 4 (CRISPR-target 1, T1), exon 6 (T2) and exon 7 (T3) were used in generating *elovl2* KOs. T1, T2 and T3 account for approximately 51%, 36% and 13% of the *elovl2* KOs used in our study. Only one *elovl2* target site was selected for each individual salmon. In addition to *elovl2*, the *slc45a2* gene involved in melanin synthesis^27^ was simultaneously targeted. CRISPR-mediated KO of *slc45a2* served as a visual marker as the phenotype of non-functional *slc45a2* is complete loss of pigmentation^26^. To study the general response of Atlantic salmon PUFA biosynthetic pathway to *elovl2* KO, the fish were fed either a “low PUFA” diet or a standard commercial diet. The low PUFA diet contained reduced levels of 20:5n-3 and 22:6n-3 but higher levels of the C_18_ precursors, 18:3n-3 and 18:2n-6, while the standard commercial diet contained relatively higher levels of 20:5n-3 and 22:6n-3 but lower composition of 18:3n-3, 18:2n-6 and 20:4n-6 (Supplemental Table 1). There were no observable differences in length and weight of fish after the feeding trial (Supplemental Table 2). Additionally, no mortality was recorded during the feeding trial.

### Confirmation and identification of *elovl2* KO Atlantic salmon

**Figure 1:**
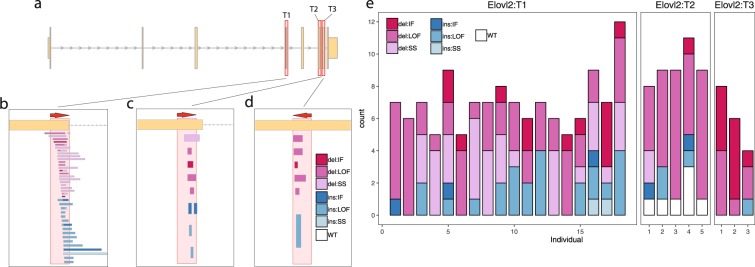
Insertions/deletions at the CRISPR target sites in the *elovl2* gene identified by Sanger sequencing for *elovl2* KOs at both 60 and 108 days of feeding. (**a**) Highlights the three CRISPR target sites (T1-3) within the coding-sequence of the *elovl2* gene. (**b**–**d**) Magnifications show the orientation of the CRISPR target site on top and the different unique indels below. (**e**) Histogram depicting the (SnpEff) predicted effects of the indels on the *elovl2* gene. Insertions/deletions are indicated in the legend by the ins/del prefix. IF: In-frame; LOF: Loss of function (indels predicted to disrupt a transcript’s reading frame or removing more than 50% of the protein-coding sequence); SS: Splice-site; WT: Wildtype.

**Figure 2:**
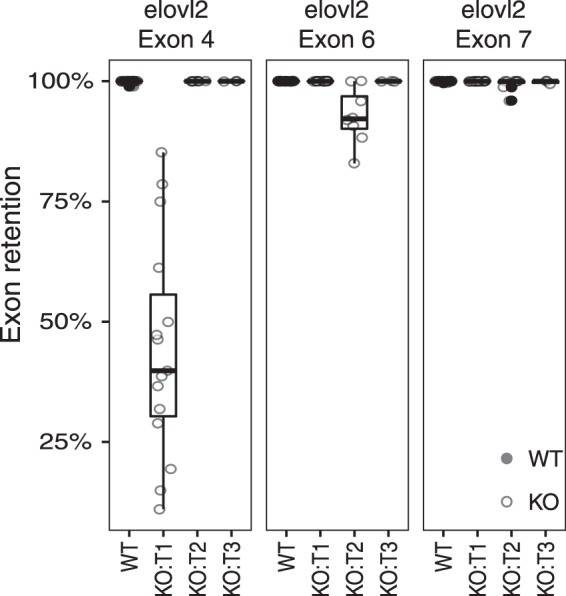
Missplicing: Exon retention for exons: 4, 6 and 7 of the *elovl2* gene. Displayed is the percentage exon retention for each individual belonging to either wildtype (WT) or one of the groups carrying a KO at target site 1–3 in the *elovl2* gene (T1-3). Only one CRISPR construct targeting either T1 or T2 or T3 was injected per individual. However, each KO individual from both time points (60 and 108 days) was analysed for exon 4, exon 6 and exon 7 retention.

CRISPR-induced mutations in the albino gene, *slc45a2* are highly correlated with indels within *elovl2* (Supplemental Table 3), thus facilitating identification of *elovl2* KOs. This high correlation was in agreement with previously reported results by Wargelius *et al*.^28^. The three different CRISPR target sites within *elovl2* gene (T1-T3) were characterised by Sanger sequencing, sequencing on average 8 clones per individual. Notably was the identification of mosaicism in albino fish containing mutant and wildtype (WT) *elovl2* alleles at T2 as well as a variety of insertions/deletions (indels) for T1-T3 (Fig. 1). Each indel was subsequently annotated using SnpEff^29^, whereby we summarised the indel effect predictions in three categories: Loss-of-function (LOF); splice-site (SS) and in-frame (IF). Both T1 and T2 were located in proximity to the intron-exon boundary of exon four and six respectively. Consequently, many of the indels, particularly those found at T1 and to a lesser degree T2 in the *elovl2* gene were predicted to affect the splicing site (Fig. 1a–c), leading to an aberrant transcript lacking exon 4. To validate these SnpEff predictions for aberrant splicing, we utilised the RNAseq data to calculate the percentage exon retention for exons 4, 6 and 7. We found abundant missplicing of *elovl2* for T1 group (Fig. 2). On average more than 50% of the *elovl2* transcripts were truncated, lacking exon 4. Additionally, we found low levels of missplicing for the T2 group whereby on average less than 10% of the transcript were truncated, lacking exon 6 (Fig. 2).

### Elovl2 KO inhibits elongation of 20:5n-3, 22:5n-3 and 20:4n-6

**Figure 3:**
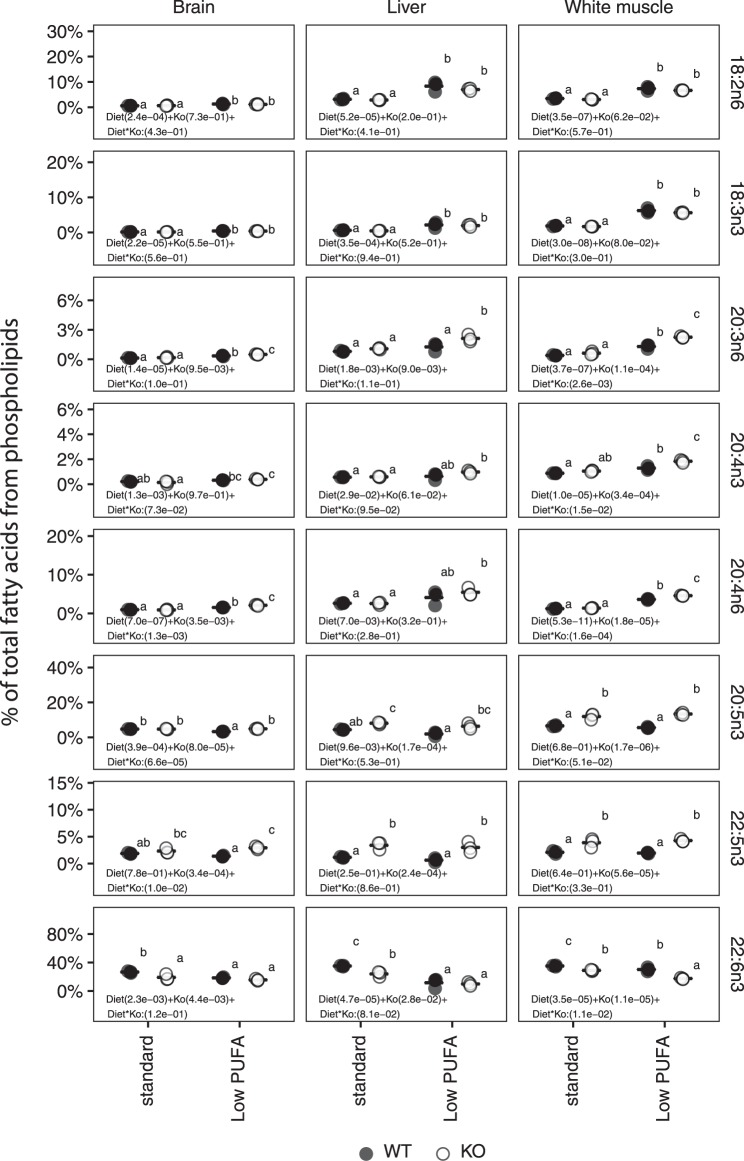
Polyunsaturated fatty acid composition of phospholipid pool in the whole Brain, liver, and white muscle of wildtype (Ctr or WT) and *elovl2* knockout (KO) fish fed either low PUFA or standard commercial diet for 108 days. Results are shown as individual data points (n = 3), the bar represents the mean value per group. ANOVA *p*-values for diets, *elovl2* KO (Ko/WT) as well as interaction (Diet*Ko) are indicated in the figure. Groups with different letters are significantly different from each other (*p*-value < 0.05; Tukey HSD test).

To investigate *in vivo* functions of *elovl2* in Atlantic salmon LC-PUFA biosynthesis, hepatic, whole brain and white muscle fatty acid composition in phospholipids was determined after 108 days of feeding trial. We observed accumulation of 20:5n-3 and 22:5n-3 in liver, brain and white muscle phospholipids in *elovl2* KO salmon compared with the WT. This was accompanied by a reduction in the levels of 22:6n-3 (Fig. 3). Similar patterns were observed in hepatic phospholipids in *elovl2* KOs compared with WT after 60 days of feeding (Supplemental Fig. 1). In addition, there was a significant (p < 0.05) accumulation of 20:4n-6 in brain and white muscle phospholipids in fish fed low PUFA diet after 108 days of feeding trial (Fig. 3). These effects were not found in liver phospholipids. Moreover, it was clear that fish fed both low PUFA and the standard commercial diets had active fatty acyl desaturation and elongation as demonstrated by accumulation of 20:5n-3 and 22:5n-3 in the liver of *elovl2* KO salmon, and relatively higher levels of 22:6n-3 in the wildtypes under the two dietary treatments (Fig. 3). The impact of *elovl2* ablation and dietary treatments on fatty acid storage was also studied by determining fatty acid composition in white muscle triacylglycerol (TAG). Similar to phospholipids, we observed accumulation of 20:4n-6, 20:5n-3 and 22:5n-3 and a reduction of 22:6n-3 in white muscle TAG in *elovl2* KO salmon compared with the WT (Supplemental Fig. 2).

### Impaired endogenous synthesis of 22:6n-3 induces hepatic mRNA expression of *srebp-1* and target genes

From the RNAseq analysis an average of 28 million reads were collected from each library, of which ~97% were mapped to the salmon genome and ~83% were uniquely mapped. Out of 55304 annotated genes in salmon genome, we found 24558 genes which passed the minimum level of expression with at least 1 count per million (CPM) in 25% of the samples. Differential expression analysis identified 19 genes (DEGs, *q* < 0.05) between KO and WT salmon fed low PUFA diet, while 6 DEGs were found in salmon fed standard commercial diet (Supplemental Table 4). Several genes involved in fatty acid metabolism were found among the DEGs, including those involved in LC-PUFA synthesis and Srebp regulation. This includes *acsl3a*, *acsl3b* and *acsl4* genes involved in fatty acid-CoA synthesis, *agpat3a* involved in phosphatidic acid synthesis and *chrac1* gene which encode a protein component of the chromatin-accessibility complex CHRAC. All of these genes were significantly (*q* < 0.05) upregulated in KO salmon (Supplemental Table 4).

**Figure 4:**
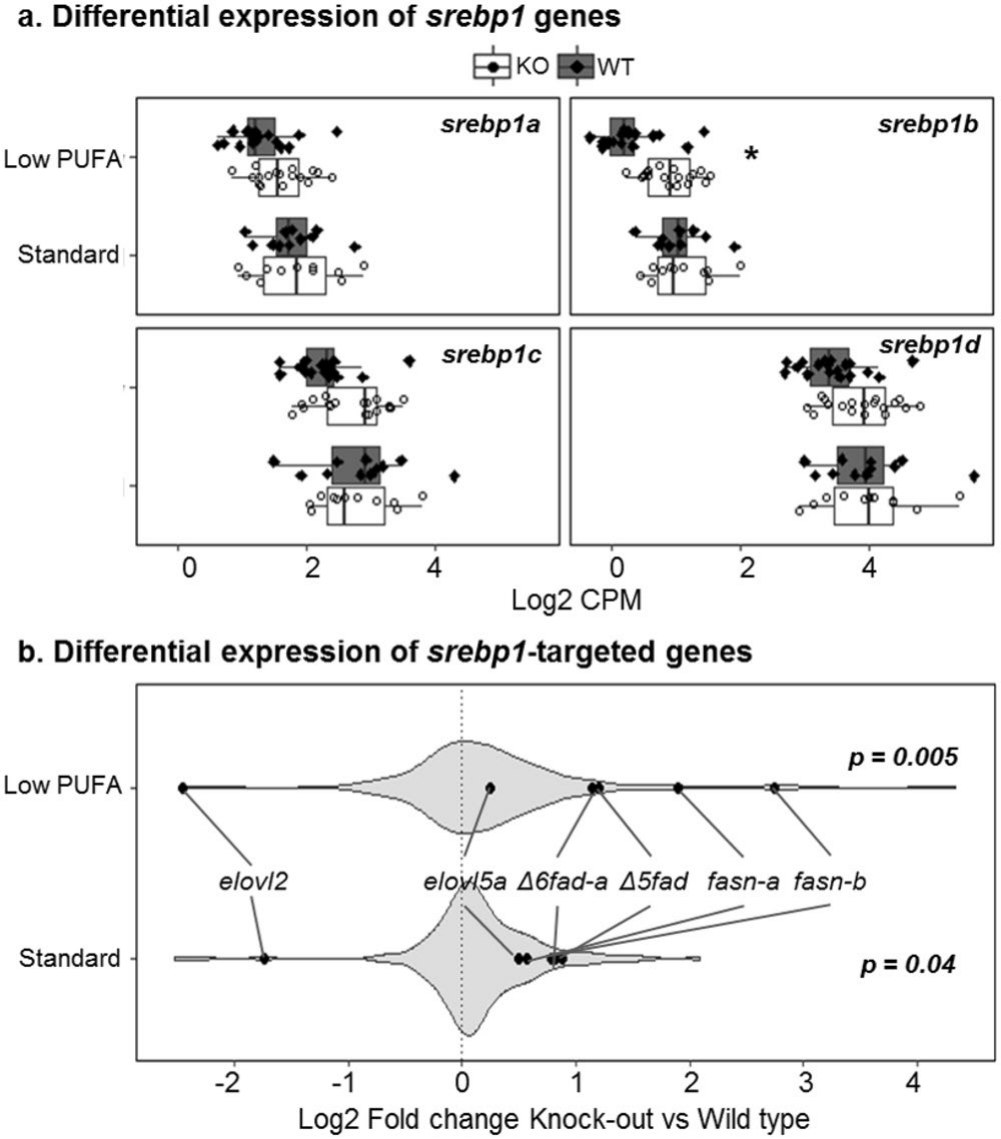
Expressional change of hepatic *srebp-1* genes (**a**) and their targeted-genes (**b**) between *elovl2* knockout (KO) and wildtype (WT) salmon fed either low PUFA or standard commercial diet. (**a**) Boxplots displaying all four *srebp-1* genes with their respective expression (log2 CPM) in KO and WT salmon fed either low PUFA or standard diet. Asterisks indicates differential expression (q < 0.05). (**b**) Violin plot showing 183 lipid genes with least 1 Srebp-1-targeted motif in the promoter region. The density of the plot is proportional to the number of genes in each log2 fold change unit. Both fish fed low PUFA or standard diet showed significant enrichment of the Srebp-1-targeted genes. Gene expression analysis was performed using n = 18 *elovl2* KO + n = 18 WT fed low PUFA diet, and n = 11 each of *elovl2* KO and WT fed standard diet.

**Figure 5:**
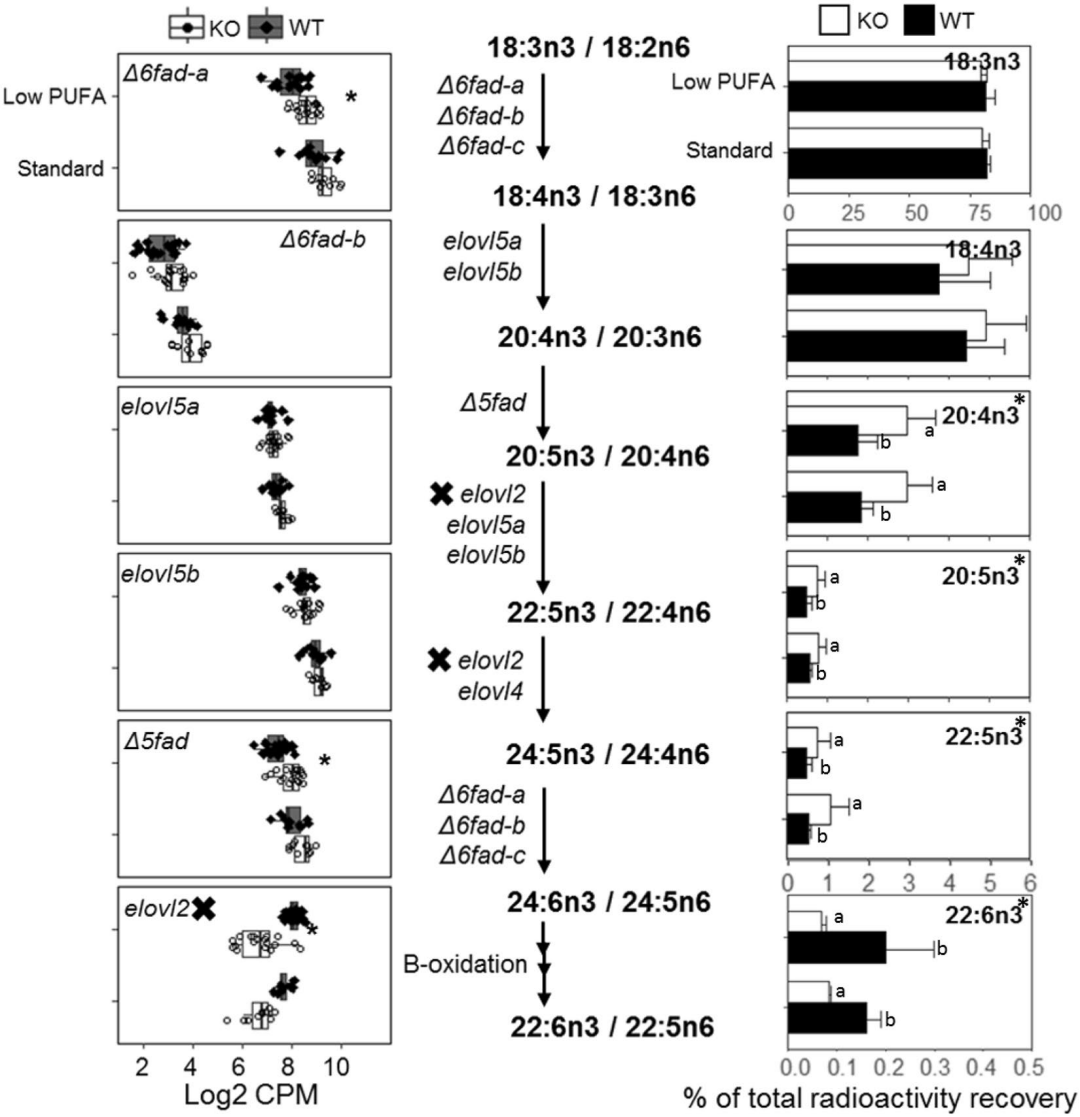
Gene expression and ^14^C fatty acids production in LC-PUFA synthesis pathway of liver between knockout (KO) and wildtype (WT) salmon. The LC-PUFA biosynthesis pathway is shown in the middle. Figures on the left indicate expression of elongase and desaturase genes in log2 count per million (Log2 CPM) between KO and WT salmon fed either low-PUFA diet (low) or standard (std) diet. Asterisks indicates differentially expressed gene (*q* < 0.05) between KO and WT salmon. The expression of *Δ6fad-c* and *elovl4* genes was not shown in the figure because their expression in liver is very low (Log2 CPM < 1). Gene expression analysis was performed using n = 18 *elovl2* KO + n = 18 WT fed low PUFA diet, and n = 11 each of *elovl2* KO and WT fed standard diet. The right figures show the percentage recovery of radioactivity in n-3 LC-PUFA from cellular total lipids after incubation 1–14C-18:3n-3 on hepatocytes from salmon fed low and std diets for 108 days. Two-way ANOVA was applied, and asterisks indicates significant (*p* < 0.05) differences in recovery of radioactivity in the fatty acid between KO and WT salmon with n = 3. No significance (*p* > 0.05) was observed between low and std diets, nor between the interactions (Diet*Ko). Statistical differences between WT and *elovl2* KO under two diets is denoted by different letters.

The expression of *Δ6fad-a*, *Δ6fad-b* and *Δ5fad* genes in LC-PUFA synthetic pathway were generally higher in KO compared to WT salmon regardless of dietary treatment while expression of *elovl2* gene was downregulated (Fig. 4b). However, the expressional differences between these genes were only significant (*q* < 0.05) when salmon was fed low PUFA diet. The expression of *elovl5a* and *elovl5b* genes was similar between KO and WT salmon according to our RNAseq data. However, RT-qPCR analysis showed slight upregulation of *elovl5a* (Supplemental Fig. 3). The expression of LC-PUFA biosynthetic genes was correlated to percentage radioactivity recovery in LC-PUFAs from isolated hepatocytes after incubating with ^14^C-18:3n-3. Our data showed significant (p < 0.05) accumulation of ^14^C-20:4n-3, ^14^C-20:5n-3 and ^14^C-22:5n-3 in hepatocytes from KO salmon compared to WT. A significant (*p* < 0.05) reduction in the levels of ^14^C-22:6n-3 in KO salmon was also observed (Fig. 5).

Additionally, we paid special attention to the expression of *srebp-1* genes, since they are well known as major transcription factors regulating fatty acid metabolism. The salmon genome contains four *srebp-1* genes at different locations (Supplemental Table 9), hereafter referred to as *srebp-1* (a–d). Expression of all *srebp-1* genes was higher in liver of KO salmon compared to WT when fed low PUFA diet, though only *srebp-1b* was significantly different (Fig. 4a). Expression of the *srebp-1* genes in salmon fed standard diet was less affected by the KO compared to the WT. To further test if *srebp-1* had a significant role in regulating gene expression, we performed gene set enrichment analysis (GSEA), testing for enrichment of 183 lipid metabolism genes with at least 1 Srebp-1 motif in their promoter region. Indeed we found that lipid genes with Srebp-1 motif tended to be significantly upregulated in KO salmon fed either low PUFA or standard diet (Fig. 4b). The effect was more distinct in the low PUFA group (*p* = 0.005 vs 0.04), which was associated to higher fold change of *fasn-a*, *fasn-b*, *elovl2*, *Δ6fad-a* and *Δ5fad* genes in low-PUFA fed salmon. Upregulation of *fasn-a* and *fasn-b* showed no observable effects on the composition of liver phospholipid 16:0 and 18:0 (Supplemental Table 10).

### RNAseq Validation

**Figure 6:**
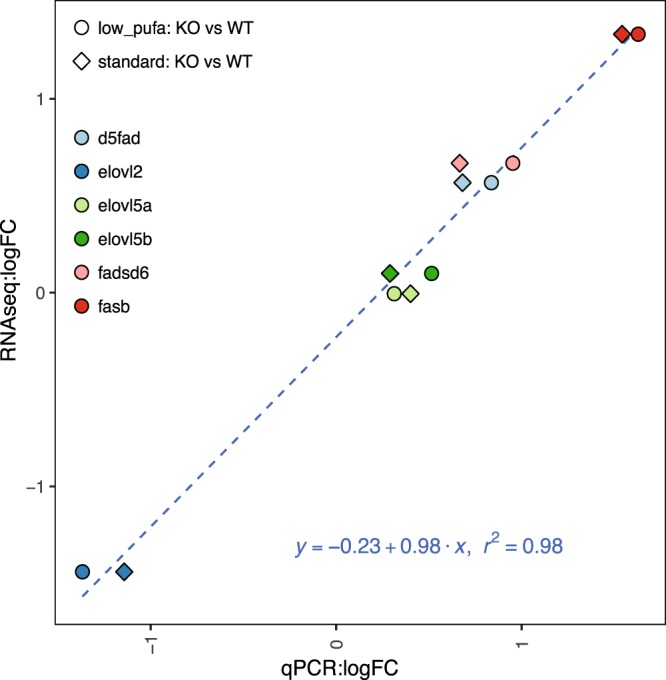
Validation of RNAseq results by RT-qPCR. Log_2_FCs from the RNAseq experiment are plotted on the y-axis, while log_2_FCs from the RT-qPCR are plotted on the x-axis. RT-qPCR data was normalized to the housekeeping gene *ef1α-b*. Expression levels of six different genes were compared. For each gene the log_2_FCs for the two contrasts: low PUFA (Ctr vs KO) and standard (Ctr vs KO) were calculated and plotted. Overall we found a highly significant correlation (p-value < 7.988e-10) between log_2_FCs from RNAseq and those calculated by qPCR. The dashed line represents the linear regression line (formula displayed in the plot).

To verify the RNAseq results, five genes were chosen and analyzed by RT-qPCR. The results showed a high, significant correlation between the log2 fold changes obtained by RT-qPCR and those obtained by RNAseq (Pearson correlation r = 0.99, p < 0.7e^−10^) (Fig. 6).

## Discussion

The ability of any species to synthesize LC-PUFAs depends on the complementary roles of fatty acyl desaturases (Fads) and very long chain fatty acyl elongases (Elovls). The *elovl5a*, *elovl5b*, *elovl2* and *elovl4* genes have been described in Atlantic salmon and their gene products functionally characterized via heterologous expression in yeast, *Saccharomyces cerevisiae*^19,20,22^. Salmon *elovl5a* and *elovl5b* encode proteins that efficiently elongate C_18_ and C_20_ PUFAs, with low activity towards C_22_^20,22^, while *elovl2* and *elovl4* effectively elongate C_20_ and C_22_ PUFAs^19,22^. Here, by generating *elovl2* KO Atlantic salmon using CRISPR/Cas9, we have shown that *elovl2* is crucial for elongation of 20:4n-6 for synthesis of longer chain n-6 PUFAs, as well as 20:5n-3 and 22:5n-3 for the formation of 22:6n-3 in multiple tissues *in vivo*. Our results are consistent with results from the heterologous studies in *S. cerevisiae*^22^, however results from the hepatocyte assay revealed a low but detectable synthesis of 22:6n-3 in *elovl2* ablated cells, suggesting a partial compensation by other elongases such as *elovl4* or *elovl5a*/*elovl5b* as these Elovl5 elongases have been shown to have some capability to elongate 22:5n-3^22^. RT-qPCR analysis showed slight upregulation of *elovl5a* in the liver and white muscle of *elovl2* KO salmon fed standard commercial diet and in liver of fish fed low PUFA diet (Supplemental Fig. 3), but this was not revealed by our RNAseq data. Furthermore, no upregulation was observed for *elovl4* (data not shown). The fact that transcriptional activity of *elovl4*, *elovl5a* and *elovl5b* are hardly affected by the *elovl2* KO does not rule out that their encoded proteins can compensate for lack of Elovl2 activity. Additionally, we cannot exclude the contribution of mosaicism to the observed background 22:6n-3 synthesis in the *elovl2* KO hepatocytes. Although sequences from 8 clones may be limiting in providing full overview of the extent of mosaicism in individual salmon, our RNAseq data showed to a reasonable degree that the mutant *elovl2* alleles are dominating. Results from the current *in vivo* study also suggest that in addition to dietary LC-PUFAs, endogenously synthesized PUFAs play important roles in transcriptional regulation of lipogenic genes in Atlantic salmon.

Truncated proteins produced by the CRISPR-induced out of frame mutations will all miss the C-terminal dilysine-motifs which are important for ER-retention. Similar frameshift mutations in human *ELOVL4* are observed in patients with autosomal dominant Stargardt-like juvenile macular dystrophy and produce truncated ELOVL4 proteins that fail to localize to the ER and lack innate condensation activity^30,31^. *elovl2* KOs generated from CRISPR-target site 3 will produce truncated proteins missing a catalytic histidine dideoxy binding motif (HVYHH) essential for fatty acid condensation. Additionally, the CRISPR-induced skipping of exon 4 will generate truncated Elovl2 proteins missing 30 amino acid residues resulting in lack of portions of a putative membrane-spanning domain^22^ and a number of conserved amino acids that will most likely render the Elovl2 protein non-functional.

Mammalian ELOVL2 is highly effective in elongation of 20- and 22-carbon PUFAs with low or no activity in the case of humans towards C_18_^32^, similar to what we observed in salmon. *Elovl2* – deficient mice displayed decreased levels of 22:6n-3 and 22:5n-6 with an accumulation of 20:4n-6, 20:5n-3, 22:5n-3 and 22:4n-6^24^. In addition to 20:5n-3 and 22:5n-3, the *elovl2* KO salmon also accumulated 20:4n-6 in brain and white muscle phospholipids as well as in white muscle TAG. Increased 20:4n-6 levels were particularly observed under low PUFA diet indicating higher conversion rate from 18:2n-6 in the feed. The levels of other n-6 PUFAs, such as docosatetraenoic acid, 22:4n-6 and 22:5n-6 was either very low or undetectable. The differences in n-6 LC-PUFA elongation between the *elovl2* KO salmon and the *Elovl2* KO mice suggest a probable preference of Atlantic salmon LC-PUFA biosynthetic pathway for omega-3 PUFAs. The rate limiting enzyme for 22.6n-3 synthesis in fish is *Δ6fad-a*, which is reported to have higher activity towards n-3 fatty acids compared to n-6 PUFAs^20,33–36^. In addition, it appears that Atlantic salmon Elovl2 has relatively lower activity towards 20:4n-6 and 22:4n-6 compared to their n-3 homologs^22^. Similarly, salmon *elovl5a* seems to have preferences towards C_18_ and C_20_ n-3 PUFAs. This does not appear to be the case for *elovl5b*^22^. Thus, it is probable that reduced activity of Δ6 Fad-a, Elovl5a and Elovl2 towards n-6 PUFA precursors decreased subsequent synthesis of longer chain n-6 PUFAs in the current study. This may have masked the impact of *elovl2* ablation on omega-6 PUFA synthesis. These observed preferences of Atlantic salmon PUFA biosynthetic pathway for omega-3 PUFAs warrants further investigation.

Atlantic salmon LC-PUFA biosynthetic pathway has been shown to be under nutritional regulation, responding to levels of dietary PUFAs^21,22,37^. Atlantic salmon fed diet containing relatively high levels of 20:5n-3 (7.3% of total fatty acids, FAs) and 22:6n-3 (10.5% of total FAs) showed reduced hepatocyte fatty acyl desaturation and elongation^37^. In fact, results from the current study suggest that fish fed low PUFA and standard commercial diet had active fatty acyl desaturation and elongation. While it is expected that the relatively high levels of 20:5n-3 and 22:6n-3 in the standard diet feed-back inhibit desaturation and elongation, the presence of appreciable levels of 18:2n-6 (10.8% of total FAs) and 18:3n-3 (4.8% of total FAs) may have had stimulatory effects on PUFA synthesis. Consistent with our observation, salmon fed diet rich in 18:2n-6 demonstrated strong fatty acyl desaturation and elongation^37^.

The brain is known to have a unique fatty acid composition with relatively high levels of 22.6n-3, 20:4n-6 and 16:0, but low levels of 20:5n-3^38,39^. Endogenous synthesis of 20.5n-3, 22:5n-3 and 22:6n-3 have been shown to be low within the brain^40,41^, suggesting that it maintains its unique fatty acid levels through uptake from dietary sources. In the present study, analysis of whole brain LC-PUFA composition in phospholipids from *elovl2* KO salmon revealed accumulation of 20:5n-3, 20:4n-6 and 22:5n-3 under low PUFA diet, suggesting a likely inhibition of elongation of these Elovl2 substrates. In contrast, whole brain phospholipid 20:5n-3, 20:4n-6 and 22:5n-3 levels remained unchanged under standard dietary condition, suggesting that acquisition of these fatty acids from the standard commercial diet was probably sufficient to meet requirements in the brain. A slight reduction in the levels of 22:6n-3 in the brain of *elovl2* KO was however noted under both dietary conditions, suggesting a reduced Elovl2 activity (Fig. 3). The *elovl2* expression in Atlantic salmon is relatively high in the brain compared to other tissues, except the intestine and liver^22^. Thus, it is compelling to suggest that the brain is capable of synthesizing LC-PUFAs under reduced dietary LC-PUFA conditions. In support of this, trout brain astrocytes incubated with ^14^C-18:3n-3 and ^14^C-18:2n-6 showed clear capacities for desaturation and elongation^42^.

Interestingly, results from the present study suggest that impaired formation of endogenous 22:6n-3 activates sterol regulatory element binding protein -1 (Srebp-1), which has been shown to be responsible for transcriptional regulation of many lipogenic genes in mice, including sterol-CoA desaturase 1, fatty acid synthase (*fasn*), *Δ6fad-a*, *elovl5*, *elovl2* and *Δ5fad*^24,43^. Accordingly, upregulation of hepatic *srebp-1*, most likely in response to the reduced levels of liver 22:6n-3 appears to induce mRNA expression of *fasn-b*, *Δ6fad-a*, *Δ5fad* and to a less extent *elovl5a* in our *elovl2* KO salmon. Additionally, GSEA of Atlantic salmon lipid metabolic genes identified the above upregulated genes as potential targets of Srebp-1 as they have at least 1 Srebp-1 binding motif within their promoter regions. Results from many studies have shown that dietary LC-PUFAs influence transcriptional regulation of Atlantic salmon *fad* and *elovl* genes^22,44–46^. In addition, gene promoter studies have reported Srebps as key transcriptional regulators of Atlantic salmon *Δ6fad-a*^46^. Thus, it appears dietary LC-PUFAs as well as endogenously synthesized 22:6n-3 regulate the expression of lipogenic genes in a Srebp-1-dependent fashion in Atlantic salmon. Consistent with our observation, Atlantic salmon SHK-1 cells supplemented with 20:5n-3 and 22:6n-3 showed decreased expression of *srebp-1* and its targets, *Δ6fad-a* and *fasn*^25^. Though it is compelling to suggest that upregulation of *srebp-1* and target genes is a response to reduced 22:6n-3 levels, we cannot exclude the possibility that it is as well a response to general impairment of the PUFA biosynthetic pathway. Taken together our lipidomics and transcriptomics data, we propose that there is a form of end-product feedback regulation of Atlantic salmon LC-PUFA biosynthetic pathway, most likely via Srebp-1 activation when end-products such as 22:6n-3 is low or inhibition when end-products are high through dietary supplementation and endogenous synthesis.

In conclusion, results from the present study highlight *in vivo* functions of *elovl2* in multiple tissues in Atlantic salmon LC-PUFA biosynthesis. Thus, our findings show the key roles of *elovl2* in elongation of 20:4n-6 for synthesis of 22:5n-6 as well as 20:5n-3 and 22:5n-3 in 22:6n-3 synthesis *in vivo*. The current study also suggests an important role of *elovl2* in 22:6n-3 synthesis in the brain of Atlantic salmon. Furthermore, our data to some extent highlights the crucial roles of endogenously synthesized PUFAs in the regulation of hepatic lipogenic genes, most likely in a Srebp-1-dependent manner. The obvious changes in the levels of LC-PUFAs in our *elovl2* KO salmon coupled with the significant hepatic transcript response, and with the fact that Elovl2 catalyses two important penultimate steps of PUFA synthesis suggests *elovl2* as a potential selective Atlantic salmon breeding marker for ensuring an increased conversion of C_18_ fatty acids present in vegetable oils to 20:5n-3 and 22:6n-3.

## Methods

### Ethics statement

All experiments in this study have been approved by the Norwegian Animal Research Authority (NARA 5741). Use of experimental animals was strictly in accordance with the Norwegian Animal Welfare Act of 19^th^ of June 2009.

### Cloning target sequences in gRNAs

For easy recognition of knockouts (KOs) and avoiding studying mosaic animals, *slc45a2* involved in melanin synthesis was simultaneously ablated with *elovl2*. The gRNA for *slc45a2* and its effect was described by Edvardsen *et al*.^26^. Using genomic and cDNA sequences for *elovl2* (NC_027318.1, NM_001136553.1), three target sites (Fig. 1a) were selected using a custom made Perl script, which identifies unique CRISPR-target sites. Candidate target sequences were screened against the latest salmon genome assembly (GCA 000233375.4) to eliminate off-target indels. Target sequences and oligonucleotides for salmon *slc45a2* and *elovl2* are listed in Supplemental Table 5. One μg of each forward and reverse oligonucleotide was annealed in T4 ligase buffer (NEB, Massachusetts, USA) by incubating at 85 °C for 10 min, followed by cooling to room temperature. One μl of diluted (1:10) annealed oligonucleotides was ligated into 50 ng of BsmBI-digested pT7-gRNA (Addgene ID# 46759)^47^ using T4 DNA ligase (NEB), and subsequently transformed into competent DH5α cells. Plasmids were prepared using QIAprep Spin Miniprep Kit (Qiagen, Germantown, USA).

### *In vitro* transcription of Cas9 mRNA and gRNA

For producing Cas9 nuclease mRNA, pTST3-nCas9n vector, codon optimized for zebrafish (Addgene ID# 46757)^47^ was linearized using XbaI (NEB) and gel-purified using Wizard® SV Gel and PCR clean-up system (Promega, Madison, USA). Cas9 mRNA was produced using the mMessage mMachine T3 kit (Ambion, Vilnius, Lithuania) and cleaned up using RNeasy Minikit spin column (Qiagen). For making gRNAs, pT7-gRNA plasmid was linearized using BamHI-HF™ (NEB) and purified using DNA Clean and Concentrator™-5 (ZYMO RESEARCH, California, USA). The gRNAs were *in vitro* transcribed using the MEGAscript T7 kit (Ambion). The mirVana miRNA Isolation Kit (Invitrogen, Bleiswijk, The Netherlands) was used to purify gRNAs. The integrity of synthesized Cas9 mRNA and gRNAs was checked using the Agilent RNA 600 Nano kit and Agilent 2100 Bioanalyzer (Agilent Technologies, Waldbronn, Germany).

### Microinjection

Atlantic salmon eggs and sperm were obtained from Aquagen (Trondheim, Norway). Eggs were fertilized with sperm in fresh water containing 0.5 mM reduced glutathione and incubated at 6–8 °C for 2–3 hours until the first cell was visible^48^. Eggs were held on concavities of plastic petri dish and injected with a mixture of 50 ng/μl gRNA each for *slc45a2* and *elovl2*, and 150 ng/μl Cas9 mRNA in Hepes buffer using the picospritzer III (Parker Automation, UK)^26,49^. Microinjected embryos were incubated at 6–8 °C until hatching. A few weeks after startfeeding, fully albino fish were sorted out, fin clipped or fully sampled to check for mutations in *elovl2*. Fin clips and tissues were stored in 100% ethanol.

### Screening for CRISPR-induced mutations

Genomic DNA was extracted from fin clips and tissues of salmon using DNeasy Blood and Tissue kit (Qiagen). Genomic targets were amplified by PCR using DyNAzyme II DNA Polymerase (Thermo Scientific, Massachusetts, USA). PCR primer sequences are listed in Supplemental Table 6. PCR products were subcloned into the pCR™4-TOPO® vector (Invitrogen) and subsequently sequenced using Bigdye™ Terminator v3.1 cycle sequencing kit (Applied Biosystems™, BLEISWIJK, NETHERLANDS).

### Feeding trials

Two diets were used in the feeding trial, a standard commercial diet (Nutra Olympic, Skretting Nutreco Company, Stavanger, Norway) containing relatively high levels of 20:5n-3 and 22:6n-3 and a low PUFA diet (SPAROS, LDA, Olhão, Portugal) with reduced levels of 20:5n-3 and 22:6n-3 but high composition of 18:2n-6 and 18:3n-3 (Supplemental Table 1). The feeding trial was carried out at the Institute of Marine Research Matre, Norway, from late January to mid-May 2017, in fresh water at 8–10 °C with oxygen saturation of 95.3% to 97.7% at outlet during the trial. All experimental fish were initially reared on the standard commercial diet to Atlantic salmon parrs of approximate weight of 20 g before feeding trial. Groups of 15 *elovl2* KOs and 15 WTs were randomly allocated to 3 tanks and fed low PUFA diet. The 4^th^ tank containing 30 *elovl2* KOs and 30 WTs was fed standard commercial diet. The lack of pigmentation in *elovl2* KOs allowed for an easy identification and distinction from WTs in the tanks. The feeding was done by automatic feeders (Arvotec single feeder). Tissues were sampled after 60 (time point 1) and 108 (time point 2) days of feeding. Fish were anesthetized with Finquel MS-222 (ScanVacc) and then killed with a blow to the head. Twelve fish per tank comprising of 6 *elovl2* KOs and 6 WTs were collected from both diets for tissue lipid analysis, gene expression studies and screening for CRISPR-induced mutations. Collected tissues were quickly frozen on dry ice and stored at −80 °C.

### Lipid analysis

Total lipids were extracted from tissues of white muscle, liver and whole brain from three fish per dietary treatment according to Folch *et al*.^50^. Total lipid content per milligram of tissue after 108 days of feeding are presented in Supplemental Table 11. Triacylglycerols and polar lipids were separated by HPTLC silica gel 60 plates (10 × 10 cm, Merck KGaA Damstadt, Germany) using hexane: diethyl ether: acetic acid (70:30:1, v/v) as developing solvent^51^. Lipid classes were visualized by exposure to iodine and then transesterified overnight at 50 °C^52^. Fatty acid methyl esters (FAMEs) were separated, identified and quantified by gas chromatography (Agilent 7890) equipped with MS (Agilent 5977B).

### Preparation of hepatocytes for assay of fatty acyl desaturation/elongation

Hepatocytes were prepared as described previously^53^, with minor modifications. Liver from three each of WT and *elovl2* KO salmon per dietary treatment was dissected, quickly perfused via hepatic vein, finely chopped and incubated with 20 ml of Solution A; Hank’s balanced salt solution supplemented with 10 mM Hepes, 1 mM EDTA and 1 mg ml^−1^ collagenase (Sigma, Missouri, USA) for 45 min at 20 °C. Digested tissues were filtered through 100 μm cell strainer (Sigma) and the cells collected by centrifugation at 400 × g for 3 min. The cell pellet was washed with 20 ml of solution A containing 10 mg ml^−1^ fatty acid free bovine serum albumin, FAF-BSA (Sigma). Thereafter, the cell pellet was washed with 20 ml freshly prepared solution B (calcium free minimum essential medium supplemented with 100 U/ml Penicillin, 100 μg/ml Streptomycin, 0.25 µg/ml Amphotericin B and pH adjusted to 7.1–7.4 by sodium bicarbonate). The cells were further purified by centrifuging at 400 × g for 30 min on top of 54% percoll solution. The hepatocytes layer were collected and washed twice with solution B.

### Incubation of hepatocytes with ^14^C-18:3n-3 and assay of fatty acyl desaturation/elongation

For each sample, 1.904 ml of hepatocytes and 96 μl of approximately 4.55 µM, 0.5 μCi ^14^C-18:3n-3 (American Radiolabelled Chemicals Inc., Saint Louis, USA) was incubated in cell culture flask at 20 °C for 2 h. The cells were isolated by centrifugation at 400 × g for 2 min and cell pellet washed with 2 ml solution B containing FAF-BSA 10 mg ml^−1^ and then homogenized in 2.5 ml of ice-cold chloroform/methanol (2:1, v/v) with 0.01% butylated hydroxyl toluene (BHT). Total cellular lipid was extracted according to Folch *et al*.^50^. Transmethylation of lipids was performed as described above. FAMEs were extracted by adding 2 ml of 2% KHCO_3_ followed by 5 ml of hexane/diethyl ether (1:1, v/v) containing 0.01% BHT. The mixture was centrifuged at 500 × g and FAMEs from the upper phase were dried under a stream of nitrogen and re-suspended in 100 μl of hexane containing 0.01% BHT. FAMEs were applied as a streak of 2–2.5 cm on 20 × 20 TLC plate pre-coated with 0.1 g/ml of silver nitrate. The plate was developed in toluene/acetonitrile (95:5, v/v) and desiccated in the dark for 30 min. Autoradiography was performed by placing the plate in an autorad cassette for 4–6 days with Kodak BioMax MR2 film and developed using Carestream Kodak GBX Developer and Carestream Kodak GBX Fixer. Bands were scraped into scintillation vials containing 2.5 ml scintillation cocktail and counted in a scintillation counter.

### RNA isolation and quality check

Total RNA from approximately 20–50 mg of liver, whole brain and white muscle was isolated using the RNeasy Plus Universal Mini Kit (Qiagen) according to manufacturer’s instructions. The integrity of isolated RNA was checked using the Agilent RNA 6000 Nano Kit and Agilent 2100 Bioanalyzer (Agilent Technologies). RNA integrity values varied from 8.3 to 10. At each time point, RNA was extracted from a total of 30 liver (15 WT + 15 KOs), 30 white muscle (15 WT + 15 KOs) and 18 whole brain (9 WT and 9 KOs) samples from salmon fed low PUFA diet. For fish fed standard diet, 12 liver (6 WT + 6 KOs), 12 white muscle (6 WT + 6 KOs) and 6 whole brain (3 WT + 3 KOs) samples were used for RNA extraction after each time point. Liver samples selected for gene expression analysis included those analysed for fatty acid composition.

### Gene expression analysis

#### Library preparation

Stranded RNAseq libraries were prepared from 1 µg total RNA from liver tissue using TruSeq Stranded mRNA library preparation kit (Illumina, San Diego, USA) using double unique indices (#20022371), according to the manufacturer’s instruction (Part 15031057 Rev.E). A total of 58 liver samples were used for library preparations: For each time point, 10–12 individuals fed standard diet (5 WT + 5 KOs at time point 1; 6 WT + 6 KOs at time point 2) and 18 individuals fed low PUFA diet (9 WT + 9 KOs). Libraries were sequenced at the Norwegian Sequencing Centre (NSC). All libraries were pooled, and the same pool was sequenced on 4 flow cell lanes on a HiSeq 3000 machine (Illumina), generating 100 bp single-end reads. RT-qPCR: To validate the RNAseq gene expression data, 1 µg of total RNA per liver, white muscle or brain sample was reverse transcribed into cDNA using the QuantiTect^®^ Reverse Transcription kit (Qiagen) following the manufacturer’s instructions. Negative controls (containing no reverse transcriptase) were included to check for genomic DNA contamination. The expression of *srebp-1* as well as fatty acid or the LC-PUFA biosynthetic enzymes, *Δ6fad-a*, *Δ5 fad*, *elovl5a*, *elovl5b* and fatty acid synthase-b (*fasn-b*) was studied by RT-qPCR using LightCycler® 96 (Roche, Munich, Germany). For fish fed low PUFA diet, liver samples from a total of 13–15 WT and 10–15 *elovl2* KOs were used for RT-qPCR analysis, whereas samples from 5–6 WT and 5–6 KOs fed standard diet were used. All primers used are reported in Supplemental Table 7. Elongation factor 1 alpha-b (*ef1α-b*), previously validated in Atlantic salmon^54^ was used as the housekeeping gene.

### Bioinformatics

#### Screening for CRISPR-induced mutations

The Sanger reads were transformed to .fastq format, residuals of the vector were trimmed off and trimmed reads were mapped to the Atlantic salmon genome (GCA 0002333375.4) using bwa^55^. Insertions/deletions overlapping with the three *elovl2* target sites were identified from the CIGAR string of the read alignments (.bam) and subsequently annotated using SnpEff^29^. Results are summarized in Supplemental Table 8.

#### RT-qPCR

Expression levels were normalised to the housekeeping gene elongation factor α-b (*ef1α-b*) using the ΔCt method. Fold changes were subsequently calculated using the ΔΔCt method^56^.

#### Analysis of RNAseq data

RNA sequencing files (.fastq) were processed in the following manner: (i) All reads from the same individual were merged into one fastq file. (ii) Reads were mapped to the Atlantic salmon genome (GenBank Accession number: GCA_0002333375.4) using STAR (v 2.6.0c)^57^. (iii) Read alignments, recorded in BAM format were subsequently used to count uniquely mapped reads per gene using featurecounts (v1.4.4)^58^, with the RefSeq gene_ids. Raw illumina reads as well as gene counts are publicly available through ArrayExpress^59^ accession E-MTAB-7220. Gene expression levels were calculated as counts per million total library counts using the R package edgeR^60^. Total library sizes were normalized to account for bias in sample composition, using the trimmed mean of m-values approach. For each dietary group (low PUFA or standard commercial diet), a differential expression analysis (DEA) was performed comparing KO to WT samples. Libraries of samples from 60 and 108 days of each dietary group were merged prior to contrasting KO vs WT in order to minimize the effect of fish development between the 60 and 108 days of feeding, as no effect on gene expression in terms of interaction between KO and sampling time point was observed. Gene set enrichment analysis (GSEA) was calculated for genes containing at least 1 Srebp motif in their promoter region (within 1.5 kb upstream of the transcription start site) using the ‘fry’^61^ function implemented within the edgeR package. Motif information for all salmon genes was available through SalmonMotifDB (SalMotifDB), a publicly accessible DB (Mulugeta T. *et al*., unpublished). In brief, SalMotifDB is a genome wide map of putative transcription factor binding sites (TFBSs) for salmonids and related fish genomes. The database contains predicted binding sites identified in the upstream promoter regions (−1000/+200 base pairs) from transcription start site (TSS) in seven fish genomes. The database was developed by scanning the promoter sequences using over 12,000 position specific scoring matrices (PSSMs) collected from different public and commercial motif databases. All candidate TFBSs were predicted by FIMO tool from MEME suite^62^. The database is accessible through a web-based platform (https://salmobase.org/shiny/3.5.0/teshmu/SalMotifDB/) for motif enrichment and analysis.

#### Exon retention analysis

Exon retention was calculated analogous to Braunschweig *et al*.^63^. In brief the percentage exon retention (PIR) was calculated as PIR = *inclusion*/(*inclusion* + *exclusion*). Whereby *inclusion* reads are defined as reads including the Exon of interest (*Eoi*); reads spanning from the adjacent 3′exon (*E3*) to the *Eoi* or from the *Eoi* to the adjacent 5′exon (E5). *Inclusion* = (*E3*-*Eoi* + *e5*−*Eoi*)/2. Exclusion reads connect *E3* with *E5*, skipping the *Eoi*. Exon spanning reads were extracted from the alignment files using a custom python script^64^ counting only exon spanning reads that were anchored by at least 8 bp on either side.

### Statistical analysis

The significance of the effects of diet and CRISPR-induced mutations on fatty acid composition and biosynthesis of LC-PUFAs from ^14^C-18:3n-3 (percentage radioactivity recovery in *elovl2* KO hepatocytes) was determined by two-way ANOVA, using dietary treatment and KO/WT as experimental factors, followed by a Tukey HSD post-tests (R version 3.5.2). Differences were reported significant when *p* value < 0.05. For RNAseq, levels of gene expression were determined as counts per million total library counts (CPM) and only genes with at least 1 CPM in more than 25% of samples were used in DEA. Genes with a false discovery rate (FDR), an adjusted p value (q) < 0.05 and absolute log2 fold change ((|Log2FC|) > 1 were considered to be differentially expressed between KO and WT. RNAseq analysis was performed in R (v3.4.2). DEA was performed using R package edgeR^60^.

## Supplementary information

Supplemental Figures and Tables

Supplementary table 4

Supplementary table 8

## Data Availability

Raw Illumina reads as well as gene counts are publicly available through ArrayExpress accession E-MTAB-7220. Additional data: Lipid, qPCR, Sanger-sequencing as well as exon retention data are available through FairDom (https://fairdomhub.org/studies/449).
