# Supplementary material for: CRISPR/Cas9-mediated ablation of elovl2 in Atlantic salmon (Salmo salar L.) inhibits elongation of polyunsaturated fatty acids and induces Srebp-1 and target genes
Source: Sci Rep. 2019 May 17;9:7533. doi: 10.1038/s41598-019-43862-8 (PMC6525179; doi:10.1038/s41598-019-43862-8)
Supplement: Supplementary file 1 — Supplemental Figures and Tables [file 41598_2019_43862_MOESM1_ESM.pdf]

CRISPR/Cas9-mediated ablation of *elovl2* in Atlantic salmon (*Salmo salar* L.) inhibits elongation of polyunsaturated fatty acids and induces Srebp-1 and target genes

Alex K. Datsomor [1], Nikola Zic [1+], Keshuai Li [1,4+], Rolf E. Olsen [1, 2+], Yang Jin [3], Jon Olav Vik [3], Rolf B. Edvardsen [2], Fabian Grammes [3], Anna Wargelius [2*], Per Winge [1*]

[1]Norwegian University of Science and Technology, Department of Biology, Trondheim, N-7491, Norway

[2]Institute of Marine Research, Bergen, NO-5817, Norway

[3]Norwegian University of Life Sciences, Department of Animal and Aquacultural Sciences, N-1432 Ås, Norway

[4]BioMar AS, Trondheim, Norway

*Corresponding authors: per.winge@ntnu.no, anna.wargelius@hi.no

[+]these authors contributed equally

Alex K. Datsomor: alex.datsomor@ntnu.no

Nikola Zic: nikolaz@stud.ntnu.no

Keshuai Li: Keshuai.Li@biomar.no

Rolf E. Olsen: rolf.e.olsen@ntnu.no

Yang Jin: yang.jin@nmbu.no

Jon Olav Vik: jon.vik@nmbu.no

Rolf B. Edvardsen: rolf.brudvik.edvardsen@hi.no

Fabian Grammes: fabian.grammes@nmbu.no

Anna Wargelius: anna.wargelius@hi.no

Per Winge: per.winge@ntnu.no

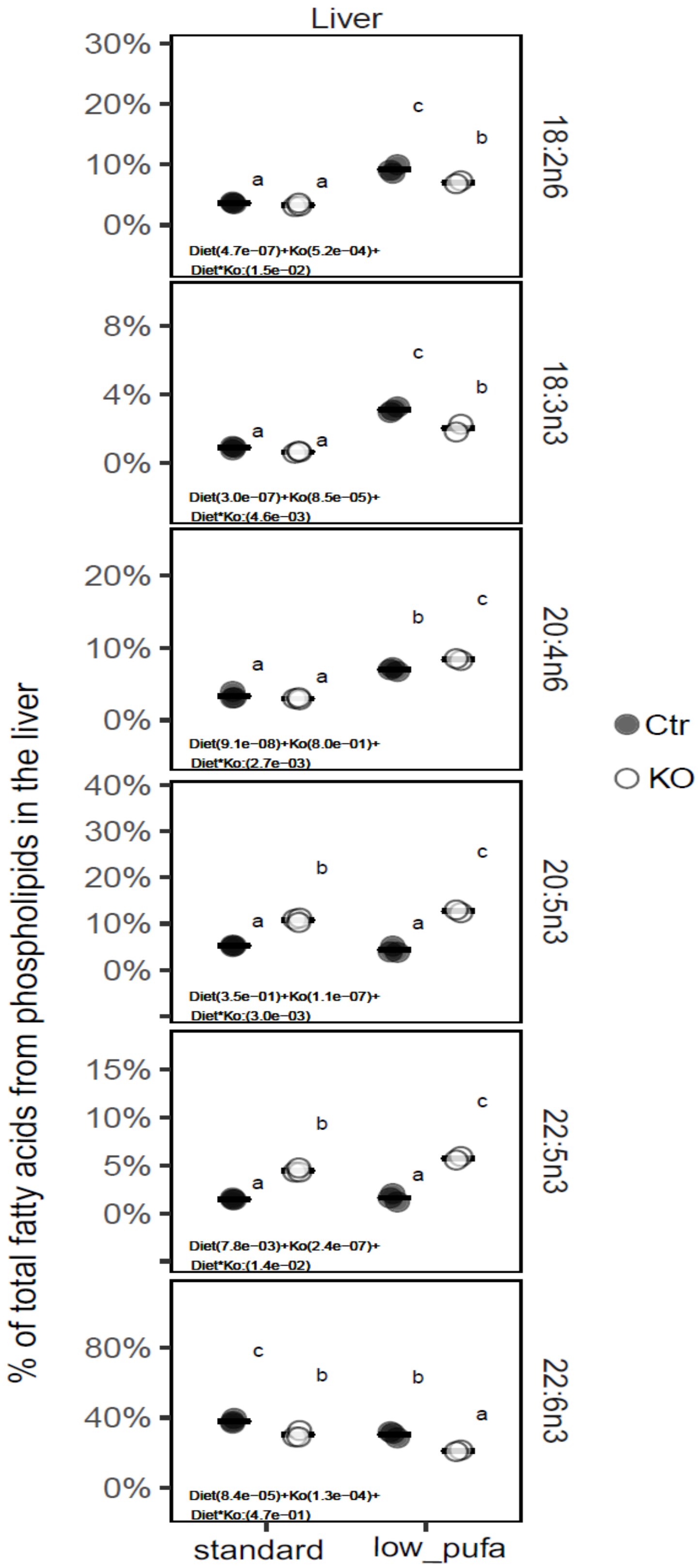

Liver
% of total fatty acids from phospholipids in the liver
18:2n6
Diet(4.7e−07)+Ko(5.2e−04)+
Diet*Ko:(1.5e−02)
18:3n3
Diet(3.0e−07)+Ko(8.5e−05)+
Diet*Ko:(4.6e−03)
20:4n6
Diet(9.1e−08)+Ko(8.0e−01)+
Diet*Ko:(2.7e−03)
20:5n3
Diet(3.5e−01)+Ko(1.1e−07)+
Diet*Ko:(3.0e−03)
22:5n3
Diet(7.8e−03)+Ko(2.4e−07)+
Diet*Ko:(1.4e−02)
22:6n3
Diet(8.4e−05)+Ko(1.3e−04)+
Diet*Ko:(4.7e−01)
standard
low_pufa
Ctr
KO

Supplemental figure 1: Polyunsaturated fatty acid composition of phospholipid pool in the liver of wildtype controls (Ctr or WT) and *elovl2* knockout (KO) salmon fed low-PUFA and standard commercial diets for 60 days. Results are shown as individual data points with n=3 for all samples except KOs under low PUFA diet with n=2, the bar represents the mean value per group. ANOVA *p*-values for diets, *elovl2* KO (Ko/WT) as well as interaction (Diet*Ko) are indicated in the figure. Groups with different letters are significantly different from each other (*p*-value <0.05; Tukey HSD test).

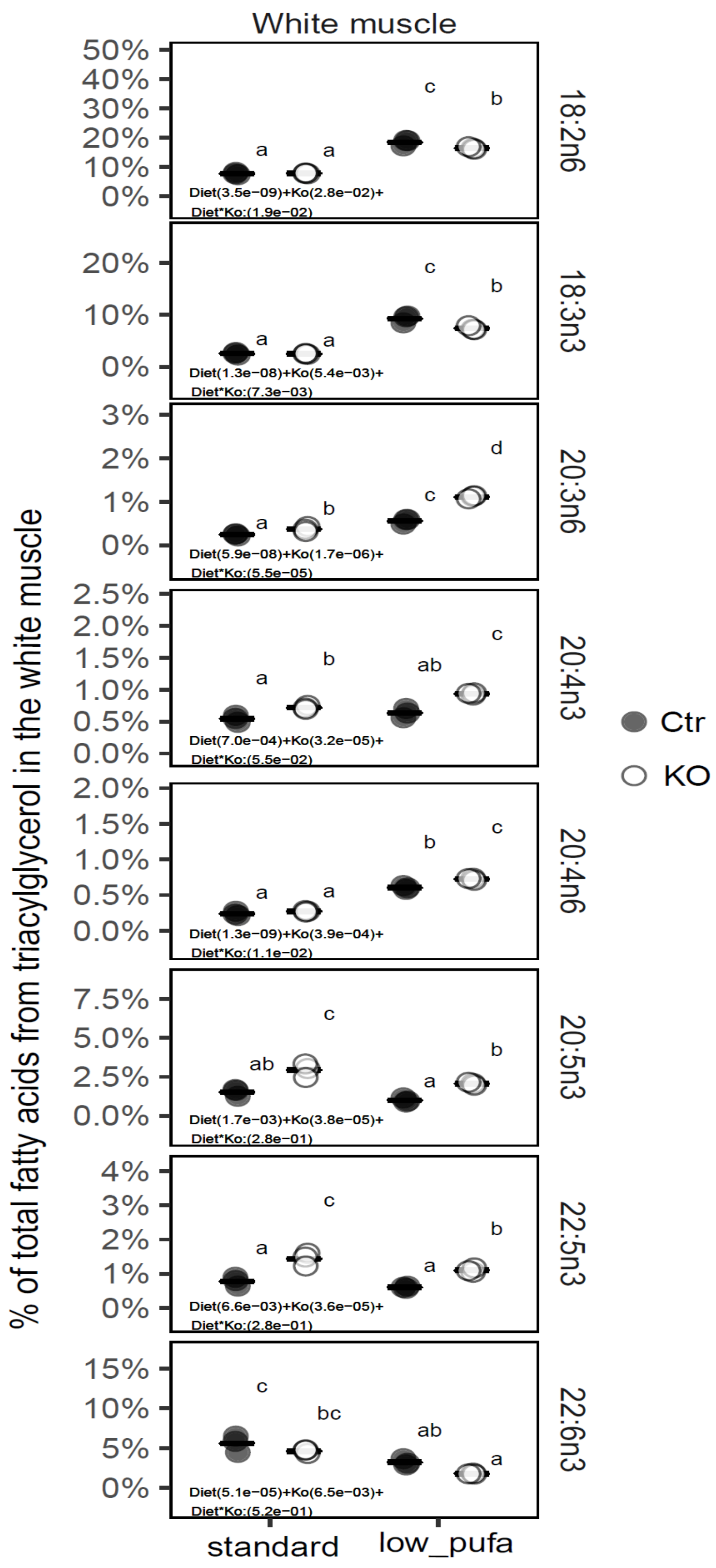
White muscle
% of total fatty acids from triacylglycerol in the white muscle
18:2n6
Diet(3.5e−09)+Ko(2.8e−02)+
Diet*Ko:(1.9e−02)
18:3n3
Diet(1.3e−08)+Ko(5.4e−03)+
Diet*Ko:(7.3e−03)
20:3n6
Diet(5.9e−08)+Ko(1.7e−06)+
Diet*Ko:(5.5e−05)
20:4n3
Diet(7.0e−04)+Ko(3.2e−05)+
Diet*Ko:(5.5e−02)
20:4n6
Diet(1.3e−09)+Ko(3.9e−04)+
Diet*Ko:(1.1e−02)
20:5n3
Diet(1.7e−03)+Ko(3.8e−05)+
Diet*Ko:(2.8e−01)
22:5n3
Diet(6.6e−03)+Ko(3.6e−05)+
Diet*Ko:(2.8e−01)
22:6n3
Diet(5.1e−05)+Ko(6.5e−03)+
Diet*Ko:(5.2e−01)
standard
low_pufa
Ctr
KO

Supplemental figure 2: Polyunsaturated fatty acid composition of white muscle triacylglycerol of wildtypes (Ctr or WT) and *elovl2* knockout (*elovl2* KO) salmons fed low-PUFA and standard commercial diet for 108 days. Results are shown as individual data points with n=3, the bar represents the mean value per group. ANOVA *p*-values for diets, *elovl2* KO (Ko/WT) as well as interaction (Diet*Ko) are indicated in the figure. Groups with different letters are significantly different from each other (*p*-value <0.05; Tukey HSD test).

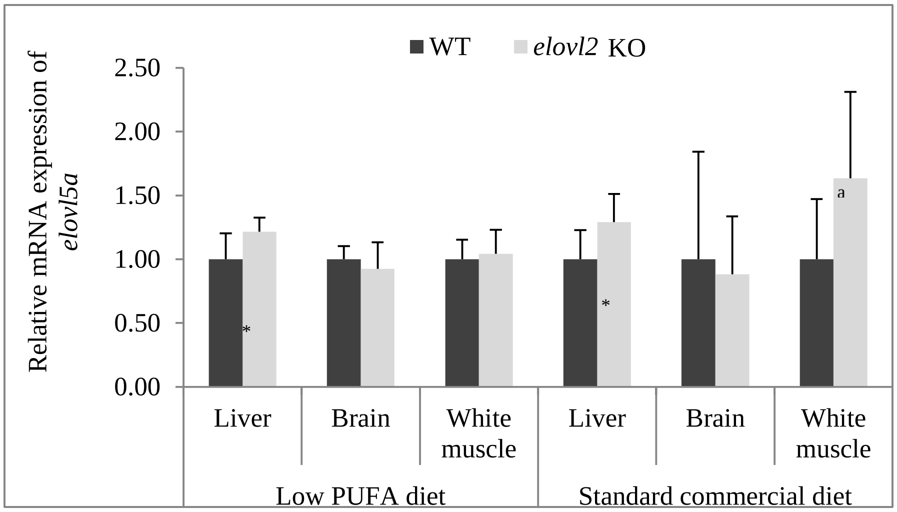

Supplemental figure 3: Relative mRNA expression levels of *elovl5a* in the liver, brain and white muscle in wildtype (WT) and *elovl2* knockout (*elovl2* KO) salmon fed low-PUFA and standard commercial diet for 108 days. Results are represented as mRNA expression in *elovl2* KO relative to wildtype (WT), with the WT set to 1. All qPCR data were analysed using qBase+ [56] which determined statistical differences between WT and *elovl2* KO using unpaired Mann-Whitney test with two-sided significance. Data are presented as means ± standard error (S.E) with n =7-13 pooled from tanks fed low PUFA and n = 3-5 for standard commercial diet. Statistical differences between WT and *elovl2* KO sampled under each diet is denoted by asterisk (*p<0.05, a = 0.056). Normalization was performed using elongation factor-1α-b (*ef1α-b*) as housekeeping gene. The expression levels of *elov5b* are not included due to high variations between biological replicates especially in the white muscle.

Supplemental table 1: Fatty acid composition (% of total fatty acids) of experimental diets

	**Low PUFA diet**	**Standard commercial diet**
**Fatty acids**	**% of total fatty acids**	**% of total fatty acids**
**Saturates**		
14:00	2.31	3.53
16:00	11.67	11.54
18:00	3.42	2.01
**Monoenes**		
16:1n7	1.91	3.16
18:1n7	2.21	2.52
18:1n9	24.48	29.7
22:1n9	0.23	6.98
**n-6 fatty acids**		
18:2n6	22.51	10.75
20:4n6	1.33	0.32
**n-3 fatty acids**		
18:3n3	17.01	4.86
20:5n3	3.78	5.4
22:6n3	2.95	7.3

Supplemental table 2: Weight and length of *elovl2* knockouts (KOs) and wildtypes (WTs) after 60 and 108 days of feeding trials

Diets	108 days of feeding trial			60 days of feeding trial		
	Experimental fish	Weight (g)	Length (cm)	Experimental fish	Weight (g)	Length (cm)
**LOW PUFA**	KO1	90	18.3	KO1	64	16.7
	KO2	102	19.4	KO2	48	15.4
	KO3	94	18.4	KO3	48	15.5
	KO4	112	19.4	KO4	81	18
	KO5	118	19.6	KO5	82	18.5
	KO6	80	17.6	KO6	81	17.5
	KO7	108	20.1	KO7	58	16.6
	KO8	108	19.4	KO8	59	16.5
	KO9	76	17.4	KO9	43	14.1
	KO10	104	19.1	-	-	-
	KO11	108	19.2	-	-	-
	KO12	nd	nd	-	-	-
	KO13	98	18.6	-	-	-
	WT1	102	19.9	WT1	59	16.6
	WT2	104	19.7	WT2	74	17.5
	WT3	88	18.2	WT3	53	16.1
	WT4	126	21.6	WT4	63	17
	WT5	130	21.6	WT5	62	16.7
	WT6	96	19.3	WT6	68	17
	WT7	78	18	WT7	90	19
	WT8	104	19.3	WT8	75	17.7
	WT9	116	21.1	WT9	66	17.5
	WT10	88	18.6	-	-	-
	WT11	144	22.1	-	-	-
	WT12	96	19.1	-	-	-
	WT13	100	20.1	-	-	-
**Standard**	KO1	100	17.8	KO1	103	19.4
	KO2	112	20.6	KO2	79	17.7
	KO3	142	21.3	KO3	91	19.1
	KO4	142	21.3	KO4	76	18
	KO5	88	18.3	KO5	82	18.2
	KO6	118	20.6	-	-	-
	WT1	148	22.7	WT1	67	17.2
	WT2	146	22.7	WT2	80	18
	WT3	150	22.3	WT3	68	17.3
	WT4	154	22.4	WT4	82	18.3
	WT5	116	20.2	WT5	71	17.3
	WT6	130	20.6	-	-	-

Supplemental table 3: Correlation between CRISPR-induced lesions in *slc45a2* and *elovl2*

Total number of albinos screened	Number of albinos with CRISPR-lesions in *elovl2*	Number of mosaics[ǂ]
46	46	8

Mosaics[ǂ]: Albino fish containing both mutant and wildtype *elovl2* allele(s)

Supplemental table 5: *slc45a2* and *elovl2* target sequences and oligonucleotides

**Name**	**Sequence**
slc45a2 target	5'-GGGGAACAGGCCGATAAGACTGG-3'**
slc45a2-F	5’-TAGGGGAACAGGCCGATAAGAC-3’
slc45a2-R	5’-AAACGTCTTATCGGCCTGTTCC-3’
elovl2 target 1	5’-GGCAGGGGAGGCGGACCTCAGGG-3’**
elovl2 target 1-F	5'-TAGGCAGGGGAGGCGGACCTCA-3'
elovl2 target 1-R	5'-AAACTGAGGTCCGCCTCCCCTG-3'
elovl2 target 2	5'-GGTGGAAGCGGTACCTGACTCAGG-3'**
elovl2 target 2-F	5'-TAGGTGGAAGCGGTACCTGACTC-3'
elovl2 target 2-R	5'-AAACGAGTCAGGTACCGCTTCCA-3'
elovl2 target 3	5’-CCCCGTCGGATGCCTGCTCTTCC-3’
elovl2 target 3-F	5'-TAGGAAGAGCAGGCATCCGACG-3'
elovl2 target 3-R	5'-AAACCGTCGGATGCCTGCTCTT-3'

** Underlined trinucleotides are CRISPR-PAM sites

Supplemental table 6: PCR primers used in target site amplification

**Name**	**Sequence**
elovl2 target 1-PCRf	5'-TGCCTCAGCTCAGTCAGTCAGG-3'
elovl2 target 1-PCRr	5'-AGAGCCCAACACAAGTGTTACT-3'
elovl2 target 2-PCRf	5'-ACTGTAGTTGTGTTGCCTTGG-3'
elovl2 target 2-PCRr	5'-ACGTAACAAAGAACATGGTCG-3'
elovl2 target 3-PCRf	5'-TAGTCTAAGAGCAACGGCCTAC-3'
elovl2 target 3-PCRr	5'-CCCCAAGGCGTTACTGTACCTG-3'

Supplemental table 7 RT-qPCR Primers

**Primer**	**Forward primer 5'-3'**	**Reverse primer 5'-3'**	**Accession no.**
srebp-1	GCCATGCGCAGGTTGTTTCTTCA	TCTGGCCAGGACGCATCTCACACT	HM561860.1
Δ5fad	GTGAATGGGGATCCATAGCA	AAACGAACGGACAACCAGA	AF478472.3
Δ6 fad-a	CCCCAGACGTTTGTGTCAG	CCTGGATTGTTGCTTTGGAT	AY458652.3
elovl5a	ACAAGACAGGAATCTCTTTCAGATTAA	TCTGGGGTTACTGTGCTATAGTGTAC	AY170327.3
elovl5b	ATTGCTAGGAGAGATGTAGGAT	GTGTGCAAAACGTTAGGAATCT	FJ237531.1
fasnb	GGTAGTGCCATACAAGTGATGT	TCAGTGGGCACCAAACATGAAC	XM_014209529.1
elf1α-b	TGCCCCTCCAGGATGTCTAC	CACGGCCCACAGGTACTG	BG933853.1

Supplemental table 9 Gene names and gene ids

genename	gene_id
elovl2	gene40749:100192341
elovl4	gene32697:106571493
elovl5b	gene51359:100192340
elovl5a	gene1745:100136433
Δ6fad-a	gene46010:100136441
Δ5fad	gene46067:100136383
Δ6fad-b	gene46066:100329172
Δ6fad-c	gene46069:106584797
fasn-a	gene51165:106589612
fasn-b	gene1567:106610271
srebp2a-a	gene7578:100502557
srebp2a-b	gene13982:106607132
srebp2b	gene13993:106607120
srebp1a	gene8199:100502556
srebp1b	gene13206:100502556
srebp1c	gene13245:106606621
srebp1d	gene13281:106606632

Supplemental table 10: Percentage of liver phospholipid 16:0 and 18:0 composition after 60 and 108 days of feeding trial with low PUFA and standard diets

		Liver phospholipid after 108 days		Liver phospholipid after 60 days	
		WT	*elovl2* KO	WT	*elovl2* KO
Low PUFA	16:0	25.6 ± 4.5	22.3 ± 1.8	16.81 ± 1.4	14.39 ± 0.6
	18:0	12.2 ± 3.0	11.7 ± 2.2	7.85 ± 0.2	8.55 ± 0.3
Standard	16:0	18.6 ± 0.9	20.3 ± 3.9	17.08 ± 0.6	16.86 ± 0.9
	18:0	5.8 ± 0.2	6.9 ± 0.8	6.20 ± 1.3	6.74 ± 0.5

Results are presented as mean ± standard deviation, with n = 3 for all samples except *elovl2* KO under low PUFA diet at 60 days feeding with n = 2.

Supplemental table 11: Lipid content per milligram of liver, whole brain and white muscle tissues after 108 days of feeding trial with low PUFA and standard diet

	**Lipid content per mg liver tissue (mg/mg)**		**Lipid content per mg white muscle tissue (mg/mg)**		**Lipid content per mg brain tissue (mg/mg)**	
	Low PUFA diet	Standard diet	Low PUFA diet	Standard diet	Low PUFA diet	Standard diet
***elovl2* KO**	0.050 ± 0.006	0.042 ± 0.004	0.024 ± 0.008	0.030 ± 0.010	0.134 ± 0.021	0.102 ± 0.053
**WT**	0.045 ± 0.003	0.038 ± 0.002	0.031 ± 0.009	0.027 ± 0.005	0.079 ± 0.022	0.099 ± 0.024

Results are presented as mean ± standard deviation of n = 3.
